# An overview of drive systems and sealing types in stirred bioreactors used in biotechnological processes

**DOI:** 10.1007/s00253-021-11180-7

**Published:** 2021-03-02

**Authors:** Cedric Schirmer, Rüdiger W. Maschke, Ralf Pörtner, Dieter Eibl

**Affiliations:** 1 Zurich University of Applied Sciences, School of Life Sciences and Facility Management, Institute of Chemistry and Biotechnology, Grüentalstrasse 14, 8820 Wädenswil, Switzerland; 2 Hamburg University of Technology, Bioprocess and Biosystems Engineering, Denickestraße 15 (K), 21073 Hamburg, Germany

**Keywords:** Bioreactor agitation, Magnetic coupling, Mechanical sealing, Lip seal, Hygienic requirements, Application trends

## Abstract

No matter the scale, stirred tank bioreactors are the most commonly used systems in biotechnological production processes. Single-use and reusable systems are supplied by several manufacturers. The type, size, and number of impellers used in these systems have a significant influence on the characteristics and designs of bioreactors. Depending on the desired application, classic shaft-driven systems, bearing-mounted drives, or stirring elements that levitate freely in the vessel may be employed. In systems with drive shafts, process hygiene requirements also affect the type of seal used. For sensitive processes with high hygienic requirements, magnetic-driven stirring systems, which have been the focus of much research in recent years, are recommended. This review provides the reader with an overview of the most common agitation and seal types implemented in stirred bioreactor systems, highlights their advantages and disadvantages, and explains their possible fields of application. Special attention is paid to the development of magnetically driven agitators, which are widely used in reusable systems and are also becoming more and more important in their single-use counterparts.

Key Points

*• Basic design of the most frequently used bioreactor type: the stirred tank bioreactor*

*• Differences in most common seal types in stirred systems and fields of application*

*• Comprehensive overview of commercially available bioreactor seal types*

*• Increased use of magnetically driven agitation systems in single-use bioreactors*

## Introduction

Stirred systems have a long tradition in biotechnological processes, especially in the biopharmaceutical industry (Birch 2010; Jossen et al. 2017; Clapp et al. 2018). It is therefore not surprising that these systems, with their distinctive agitators, are being increasingly used to primarily reduce inhomogeneities in fluids through mixing, and thus improve product quality, increase chemical and biological turnover, and accelerate heat and mass transfer (Pahl 2002; Meyer et al. 2016). Their fields of application range from non-sterile applications with microorganisms lasting a few days to axenic long-term processes with plant, animal, and human cell cultures (Table 1). For the microbial (including yeasts) production of biofuels, copolymers, and other bioproducts, non-sterile open fermentation processes are used, which can range from a few days to processes that run continuously for months (Li et al. 2014). However, batch or fed-batch processes mainly using *Escherichia coli* that last only a few hours or days dominate in the microbial field (Terpe 2006; van Heerden and Nicol 2013; Li et al. 2014). This contrasts with mammalian cell-based products, such as therapeutic antibodies, enzymes, hormones, and stem cell therapeutics, that usually involve fed-batch or perfusion processes lasting several days or weeks making them very expensive to produce (Meyer and Schmidhalter 2014; Bausch et al. 2019; Haigh et al. 2020). Therefore, compliance with good manufacturing practices (GMP) and the absence of contaminants are of utmost importance (Haigh et al. 2020). In addition to animal cell culture, where Chinese hamster ovary cells (CHO) are still dominant, processes with insect and plant cells and open and closed production processes with algae are well established (Meyer and Schmidhalter 2014).

**Table 1:** Vulnerability to contamination (from low −− to high ++ ) of different cell lines, and typical duration of different process modes

Organism (example)	Vulnerability to contamination	Batch	Fed-batch	Continuous/perfusion
Bacteria (*E. coli*)	−− to +	Hours [A, B]	Hours-days [C, D]	Weeks-months [E,F,G]
Yeast (*S. cerevisiae*)	−− to +	Hours-days [H,I]	Hours-days [J,K]	Days-months [J,L]
Algae (*C. zofingiensis)*	−− to +	Days-weeks [M,N]	Weeks [O,P]	Weeks [M,O]
Plant (*N. tabacum*)	o to +	Weeks [Q, R]	Weeks [S]	Weeks [Q,T]
Insect (*Sf*-9)	+	Days [U, V]	Days-weeks [V,W]	Weeks [V, X]
Mammalian (CHO)	++	Days-weeks [Y,Z]	Weeks [AA,AB]	Weeks-months [AA,AC]
Stem cells	++	Days-weeks [AD]	Weeks [AD,AE]	Days-weeks [AF,AG]

[A] (Hausjell et al. 2018), [B] (Schirmer et al. 2017), [C] (Kante et al. 2018), [D] (Korz et al. 1995), [E] (Tosa et al. 1974), [F] (Unrean and Srienc 2010), [G] (van Heerden and Nicol 2013), [H] (Bruder et al. 2016) ,[I] (Scheiblauer et al. 2018), [J] (Mohd Azhar et al. 2017), [K] (Arshad et al. 2017), [L] (Li et al. 2014), [M] (Benvenuti et al. 2016), [N] (Travieso Córdoba et al. 2008), [O] (Liu et al. 2014), [P] (Sun et al. 2020), [Q] (Lee and Kim 2006), [R] (Holland et al. 2013), [S] (Schiel et al. 1984), [T] (Lee et al. 2004), [U] (Imseng et al. 2014), [V] (Jardin et al. 2007), [W] (Elias et al. 2000), [X] (Akhnoukh et al. 1996), [Y] (Trummer et al. 2006), [Z] (Brunner et al. 2017), [AA] (Bausch et al. 2019), [AB] (Möller et al. 2020), [AC] (Vogel et al. 2012), [AD] (Jossen et al. 2014), [AE] (Jossen et al. 2016), [AF] (Abecasis et al. 2017), [AG] (Simaõ et al. 2016)

The vulnerability of bacteria, yeast and algae depends on the production process (e.g., biofuel production processes have a relatively low vulnerability in comparison to biopharmaceutical production processes). For reasons of clarity, the literature sources have been summarized below the table

The wide range of available production organisms and processes imposes different requirements on stirred bioreactors. For this reason, the main elements of reusable and single-use stirred bioreactors and the most frequently used drive systems, including a special focus on seals, will be summarized and discussed in terms of their suitability for various processes. In addition, the trend towards increased use of magnetic driven agitators will be discussed, and a decision tree for selecting suitable seal types for use in stirred bioreactors in biotechnological processes will also provide for the reader. Finally, emerging developments concerning popular single-use technology will be presented.

## Stirred bioreactors and their main components

The wide acceptance and frequent use of stirred bioreactors can be attributed to the early standardization of stirred systems and the introduction of hygienic design principles, work on which was begun in 1982 by the German Society for Chemical Engineering and Biotechnology (DECHEMA) and is still continuing today (DECHEMA 1982; DECHEMA 1991; ASME 2019). In addition, extensive investigations of transport processes, power input, and fluid dynamics based on experimental methods and computational fluid dynamics (CFD) have also been carried out, which have significantly influenced the geometric specifications of vessel designs, as well as the use and configuration of a large variety of different impellers and other components such as baffles and probes (Liepe et al. 1998; Nienow 1998; Zlokarnik 2001; Hemrajani and Tatterson 2003; Mirro and Voll 2009; Zhong 2010; Zhu et al. 2013; Werner et al. 2014; Meusel et al. 2016; Schirmer et al. 2018). Furthermore, recommendations for the biological evaluation of bioreactor performance for different processes (Adler and Fiechter 1983; Wagner 1987; Schirmer et al. 2017; Schirmer et al. 2019) as well as different scale-up strategies have also been successfully established (Junker 2004; Zlokarnik 2006; Catapano et al. 2009; Garcia-Ochoa and Gomez 2009).

In addition to the actual design of the vessel and its peripheral elements, magnet-driven and direct-driven options have also been developed for the agitator in the vessel that are specific to certain fields of application. However, increased attention must be paid to hygienic design in order to minimize the risk of contaminating the product and/or the environment when using direct-driven systems (Menkel 1992; Wegel and Heine 1996; Hinrichs et al. 2018). As a result, and in an effort to minimize such sterility concerns, single-use technology is increasingly being used in the production of high-value products (Haigh et al. 2020).

## Reusable vs. single-use

The growing acceptance of single-use bioreactor systems made of plastics, which are increasingly used as alternatives to gold standard stainless steel systems, especially in the biopharmaceutical industry (Eibl et al. 2018; Jossen et al. 2019; Werner et al. 2019), can be explained by the technical requirements and durations of cell culture processes. In mammalian cell culture processes, a good hygienic design concept and the avoidance of potential (cross-)contamination are essential, which can be achieved more easily by using single-use bioreactors. Thus, these systems, if correctly selected and handled, are safer, more flexible, smaller, cheaper, and greener than their reusable counterparts. These advanatges outweigh any limitations, such as leaks, breakage, leachables, and extractables. Furthermore, pre-sterilized systems can be put into operation much faster, since time-consuming and expensive cleaning and heat sterilization are eliminated. For microbial processes, the limitations are usually due to insufficient mixing, oxygen supply, or heat transport, which can often still only be overcome through the use of stainless steel bioreactors. Therefore, the growing market share of single-use bioreactors can only be explained by the focus on mammalian cell cultures in biopharmaceutical production processes (Jossen et al. 2017; Eibl and Eibl 2019; Haigh et al. 2020).

## Main components of stirred bioreactors

**Fig. 1:**
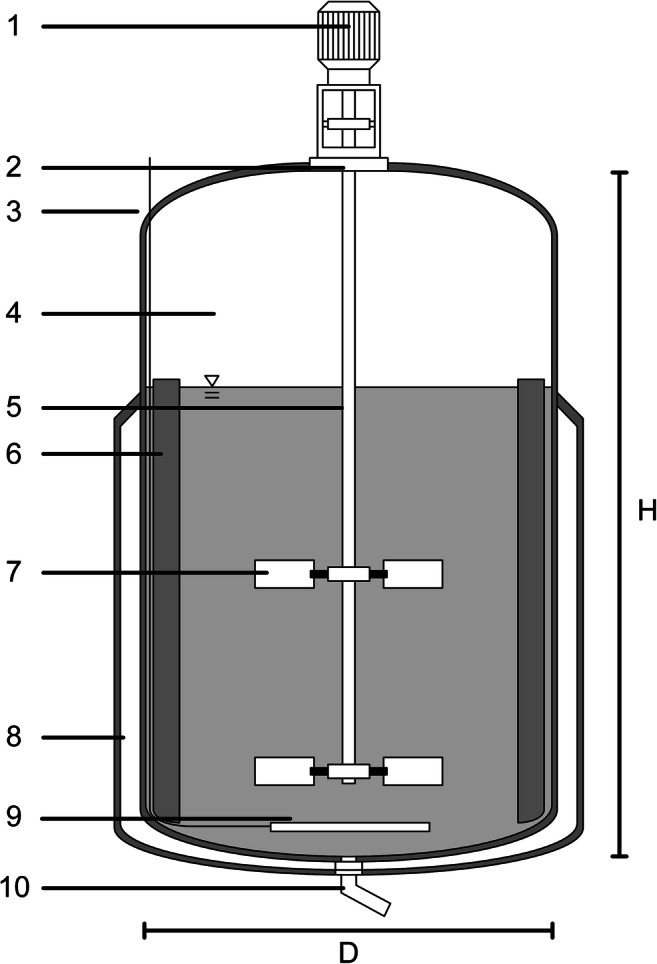
Classical composition of a stirred tank bioreactor (4) equipped with a motor (1), mechanical seal (2), air inlet (3), shaft (5), baffles (6), impellers (7), double jacket for heat transfer (8), sparger (9) and bottom drain (10). H, vessel height; D, vessel diameter

A conventional stirred bioreactor consists of a vessel equipped with a motor, a shaft with impellers on it, an air inlet, and a bottom drain (Fig. 1). The vessel is usually cylindrical, although square or rectangular vessels are possible (Hemrajani and Tatterson 2003; Nienow et al. 2016). The bottoms or lids are either flat or hemispherical, with a dished bottom being the most common type. This provides increased pressure resistance compared to planar forms and results in a lower height than hemispherical elements. Avoiding edges and dead zones in the connection between the bottom and the vessel wall facilitates cleaning and has a positive effect on the fluid flow pattern. In contrast, a flat lid would be used if the bioreactor is located in a room with limited overall height or to improve accessibility for the installation of probes, corrective devices, and additional feed streams; however, horizontal surfaces should be avoided for hygienic design reasons (Gleich and Weyl 2006; Nienow et al. 2016; Hinrichs et al. 2018). An important characteristic of stirred bioreactors is the height to diameter (H/D) ratio, which varies depending on the application. While in the chemical industry, for example, a ratio of 1:1 is typical, a ratio of 2:1 is preferred for cell culture bioreactors at laboratory and pilot scales. For microbial systems, values of 3:1 dominate since this leads to longer residence times for supplied gases, such as air or oxygen, and better temperature control due to the larger surface to volume ratio (Menkel 1992; Jossen et al. 2017; Clapp et al. 2018). Nevertheless, as bioreactor size increases, H/D ratios of 5:1 (Chisti 2006) and up to 6:1 (Najafpour 2015) can also be found. An example of a H/D ratio of 5:1 is the Thermo Scientific HyPerforma Single-Use Bioreactor (Thermo Fisher Scientific Inc. 2019), where the 5:1 ratio creates a better turn down ratio. Furthermore, the vessels are normally equipped with a gassing device (sparger), heat transfer surfaces, a bottom drain, wall baffles, and sometimes draft tubes. In case of centrically mounted impellers, baffles prevent the rotation of the liquid volume and, by creating additional turbulence, cause axial mixing between the top and the bottom of the tank (Hemrajani and Tatterson 2003; Jossen et al. 2017). The most important element is the stirring system, as it transfers the energy required for the mixing process to the fluid. It usually consists of an agitator shaft with one or more impellers on it that is inserted into the vessel through a sealed hole in the top or bottom, with the motor located outside the bioreactor (Menkel 1992; Hemrajani and Tatterson 2003; Reichert et al. 2012; Chmiel and Weuster-Botz 2018). Differences between impeller types will not be discussed in detail in this review, since this has been well described elsewhere (Liepe et al. 1998; Zlokarnik 2001; Nienow 2010; Buffo et al. 2016; Scargiali et al. 2017). However, there are some vital aspects to consider when selecting an impeller, such as the type, number, and arrangement of the impellers on the shaft, which may limit the possible application of certain seal types and influence the seal design. Based on the flow pattern, impellers can be divided into axial and radial conveying impellers (Kumaresan and Joshi 2006; Buffo et al. 2016; Zhang et al. 2017; Clapp et al. 2018). Radial pumping impellers, the most common type of which is the Rushton turbine (Nienow 2010), produce a horizontal flow. These are typically used at high speeds with high gassing rates in microbial cultivations to ensure proper mixing, high oxygen input, and good heat exchange. Axial pumping impellers, such as the 3-blade segment impeller, generate a vertical flow field, which can be further divided into upward and downward conveying agitators. The main field of application for axial impellers is animal cell culture processes, where gentle mixing and avoidance of sedimentation at low speeds and low gassing rates are a priority (Jossen et al. 2017). Due to the various process requirements and resultant differences in rotational speeds, and thermal and mechanical loads, the shaft seal is considered a critical element for guaranteeing sterile operations.

## Drive systems and seals commonly used in biotechnological processes

### Drive systems and their locations

A conventional drive system consisting of a motor and a gear or a motor coupled directly to the stirrer shaft can be mounted on the top, the side, or below the vessel, depending on the mixing task and vessel geometry. Economic and process engineering considerations also have a decisive influence on the positioning of the drive system (Menkel 1992; EKATO Holding GmbH 2012). Top drives are most common and thus the standard solution for vertical cylindrical vessels and small-scale bioreactors (Menkel 1992; Raj and Karanth 2005; EKATO Holding GmbH 2012). However, this makes accessibility to the headspace for additional ports and the removal of the lid more difficult (Menkel 1992). For economic reasons, sideways installation is often used for large storage tanks, with several small agitators usually installed to allow for variations in liquid level. In this case, the demands on the sealing technology are considerably higher than for a top drive (Jagani et al. 2010; EKATO Holding GmbH 2012). In contrast to top drives, much shorter and thus thinner agitator shafts can be used for bottom drive systems, since the effective bending moments are smaller (Creathorn 2003). This also means that there is no need for additional wear-prone shaft bearings inside the vessel (Chisti 2010; EKATO Holding GmbH 2012). Routing the shaft through the bottom of the vessel also allows impellers to be installed lower in the tank, thus reducing the minimum agitated liquid level. However, in contrast to top-driven systems, the sealing elements are exposed to chemical, biological, and abrasive loads (Menkel 1992; Jagani et al. 2010), which result in increased periodic maintenance and shorter replacement intervals (EKATO Holding GmbH 2012). Agitators are mostly arranged centrically in both top- and bottom-driven systems (Jagani et al. 2010; Clapp et al. 2018). It should be noted that in order to avoid vortex formation and to ensure proper mixing, drives can also be installed eccentrically or mounted at an angle so that baffles can be avoided (Penicot et al. 1998; Assirelli et al. 2008; Jagani et al. 2010; Clapp et al. 2018). In addition to the selection of the motor installation location, application and process-related factors also play a decisive role when selecting the most appropriate seal type, which will be discussed in the following subsection.

### Seal types: an overview

Irrespective of the installation method, the driving force of the motor must ultimately be transmitted to the fluid. In the majority of cases, this is performed via the agitator shaft, which is connected to both the non-sterile environment and the process room, sterile, or axenic environment inside the vessel. For this reason, it is important to seal the interface between the stirred tank bioreactor and the stirrer shaft in a manner that is compatible with the required operating conditions. Therefore, different sealing principles are implemented based on temperature, pressure, speed, and sterility requirements. Aseptic seals need to be able to prevent contamination in both directions by stopping undesirable microorganisms from entering the medium or liquids from leaking out of the vessel (Menkel 1992; EKATO Holding GmbH 2012). The sealing elements in reusable systems for biopharmaceutical applications must also be suitable for performing cleaning-in-place (CIP) and sterilization-in-place (SIP) procedures (ASME 2019).

As a consequence of the rotation of the shaft, it is necessary to use radial or axial dynamic seals (Hinrichs et al. 2018). Radial seals, such as radial shaft seals, lip seals, and stuffing boxes, rely on radial forces acting on the seal within an axially aligned sealing gap, meaning they are unaffected by axial forces. However, radial forces can lead to leakage and rapid wear due to radial shaft distortions. In contrast, axial seals, such as mechanical seals, act on a horizontal sealing surface, meaning they are unaffected by radial shaft deflections. Nevertherless, axial displacements can lead to leakages and must be taken into account in the seal design. Hermetic sealing is a fundamentally different approach, in which the force is indirectly transmitted from the external motor to the impeller in the vessel using magnetic coupling (EKATO Holding GmbH 2012).

The advantages and disadvantages of the individual seal types are summarized in Table 2. There is a niche for each type: stuffing boxes, which are the cheapest and simplest, are used for non-sterile applications in microbial or algae-based biomass processes; simple and inexpensive lip seals are used for small systems with minor sterility issues and a short service life (e.g., small-scale single-use bioreactors for non-GMP processes); mechanical seals are an all-round solution that are particularly effective at high speeds; and magnetic couplings are suitable for highly sterile scenarios and scenarios where containment is an issue. However, the use of different seals and agitator coupling methods is also influenced by factors such as motor position, bioreactor material, and the cells or microbials involved.

**Table 2:** Comparison of typical operation conditions for various seals, with scaling from very good: ++, good: +, average: o, and comparatively poor: -, n.a.: not available

	Stuffing box	Lip seal	Mechanical seal	Magnetic drive
Max. pressure in bar	300	6	450	> 400
Max. temperature in °C	520	100	450	120
Sliding speed in m/s	0.3	35	100	(torque dependent)
Scale	Small to large	Small	Small to large	Small to large
Shaft diameter in mm	10–200	n.a.	5–500	n.a.
Sterility	-	o	+	++
Motor location	Top	Top	Top/bottom	Top/bottom
Lifetime	o	o	+	++
Price	low	low	medium	high

To evaluate the practical application of these seals, which are discussed in the following subchapters, in everyday biotechnological operations, numerous “off the shelf” bioreactor models are examined (Table 3). The focus is liter-scale bioreactors (benchtop and pilot scale), since larger industrial-scale plants are usually custom-built, and the available data is correspondingly limited.

**Table 3:** Overview of available bioreactor systems with their field of application, motor position and sealing types

Supplier	V_max_	Motor position		Material		Application		Sealing		
Product line	[L]	Top	Bottom	SU	RU	MO	CC	MS	MC	LS
Applikon										
MiniBio	0.8	+	-	-	+	+	+	-	-	+
AppliFlex ST	3	+	-	+	-	+	+	-	-	+
eZ Glass	16	+	-	-	+	+	+	o	+	+
BioBench	22.5	+	-	-	+	+	+	-	+	-
Pilot Cell	100	+	-	-	+	-	+	o	+	-
Pilot Microbial	100	+	+	-	+	+	-	o	+	-
Belach Bioteknik										
Greta	1	-	+	-	+	+	-	-	+	-
Ant	5	+	-	-	+	+	-	-	-	+
Dolly	6	+	-	-	+	+	-	+	-	-
Hanna	10	+	-	-	+	+	+	-	-	+
Lars	30	-	+	-	+	+	+	-	+	-
Gustav	5000	o	+	-	+	+	-	-	+	o
Bilfinger										
Labqube	100	-	+	-	+	+	+	-	+	-
Pilotqube	1000	-	+	-	+	+	+	-	+	-
BioEngineering										
KLF	2.5	-	+	-	+	+	+	+	o	-
Ralf	4.5	+	-	-	+	+	+	+	o	-
NLF	20	o	+	-	+	+	+	+	o	-
Bionet										
F0	5	+	-	-	+	+	+	+	-	-
F1	10	+	-	-	+	+	+	+^c)^	-	-
F2	30	+	-	-	+	+	+	+^c)^	o	-
F3	200	+	o	-	+	+	+	+^c)^	o	-
Broadley James										
Bioreactor	16	+	-	-	+	+	+	-	o^a)^	+
CerCell										
CellVessel	27	+	+	+	-	-	+	+	-	-
BactoVessel	27	+	+	+	-	-	+	+	-	-
Cleaver Scientific										
proSet A	20	+	-	-	+	-	+	+	-	-
proSet B	20	+	-	-	+	+	-	+	-	-
proSet D	10	+	-	-	+	+	-	+	-	-
CSFS	1000	+	-	-	+	+	-	+	-	-
Cytiva (formerly GE Healthcare)										
Xcellerex XDRMO	500	-	+	+	-	+	o	-	+	-
Xcellerex XDR	2000	-	+	+	-	-	+	-	+	-
Distek										
BiOne	5	+	-	-	+	-	+	-	+	-
Electrolab										
FerMac 310/60	21	+	-	o^d)^	+	+	+	-	o	+
FerMac 320	18	+	-	o^d)^	+	+	+	-	o	+
Eppendorf										
BioBlu c	40	+	-	+	-	-	+	-	+	-
BioBlu f	3.75	+	-	+	-	+	-	-	+	-
DASbox Mini BR	0.25	+	-	-	+	+	+	-	-	+
DASGIP Bioblock	1.8	+	-	-	+	+	+	-	-	+
DASGIP Benchtop	3.8	+	-	-	+	+	+	-	-	+
BioFlo	10.5	+	-	-	+	+	+	+	o	-
CelliGen 510	32	+	-	-	+	-	+	+	-	-
Frings										
PROREACT B	1000	-	+	-	+	+	-	+	-	-
PROREACT P	1000	-	+	-	+	+	-	+^e)^	-	-
Infors HT										
Multifors 2	1	-	+	-	+	+	+	-	+	-
Minifors 2	4	+	-	-	+	+	+	+	-	-
Labfors 5	10	+	-	-	+	+	+	+	+^b)^	-
Techfors	30	+	-	-	+	+	+	+	+^b)^	-
Lambda										
Minifor	6	+	-	-	+	+	+	-	-^f)^	-
MDX Biotechnik										
MDX	10	+	-	-	+	+	+	-	+	-
Merck										
Mobius CellReady	2.4	+	-	+	-	-	+	-	-	+
Mobius CellReady	500	-	+	+	-	-	+	-	+	-
Pall										
Allegro STR	2000	-	+	+	-	-	+	-	-	+
iCellis	70	-	+	+	-	-	+	-	+	-
Pierre Guerin Technologies										
Primo	10	+	-	-	+	+	+	+	+	-
BioPro Evo	50	+	-	-	+	+	-	+	+	-
BioPro Lab & Pilot	300	+	+	-	+	+	+	+	+	-
Nucléo	1000	+	-	+	-	-	+	-	+	-
Solaris										
Black Jar	30	-	+	+	-	-	+	-	+	-
M Series	145	-	+	-	+	+	+	+	-	-
Sartorius										
Ambr 250	0.25	+	-	+	+	+	+	-	-	-
UniVessel SU	2	+	-	+	-	-	+	-	-	+
UniVessel Glass	10	+	-	-	+	+	+	+	-	-
Biostat Cplus	30	+	-	-	+	+	+	+^c)^	-	-
D-DCU	200	-	+	-	+	+	+	+	o^b)^	-
Cultibag STR	2000	+	-	+	-	-	+	-	+	-
Sysbiotech										
Pro-Lab	10	+	-	-	+	+	+	+	+	-
Pilot	70	+	+	-	+	+	+	+	+	-
Thermo Fisher										
HyPerforma Glass	10	+	-	-	+	+	+	+	-	-
HyPerforma S.U.B.	2000	+	-	+	-	-	+	-	-	+
HyPerforma S.U.F.	300	+	-	+	-	+	-	-	-	+
HyPerforma DynaDrive	5000	+	-	+	-	-	+	-	-	+
2mag										
bioReactor	0.015	-	+	+	-	+	o	-	+	-

^a^For *V*_max_ up to 3.5 L; ^b^for cell culture version, limited stirring speed; ^c^double mechanical seal as option for cell culture; ^d^utilizing CerCell vessels; ^e^double mechanical seal; ^f^utilizing vibromixing technology

Maximum working volume: *V*_*max*_, standard option; +, optional; o, not feasible; -, *SU*, single-use; *RU*, reusable; *MO*, microorganisms; *CC*, cell culture, *MS*, mechanical seal; *MC*, magnetic coupling; *LS*, lip seal. The focus is on laboratory and pilot bioreactors. If the systems with the same design also exist in m^3^ scale, the largest possible working volume is given

First of all, it can be stated that magnetic and mechanical couplings are used more or less equally. Many manufacturers offer both a direct, mechanically sealed connection and a magnetic coupling as an option for their bioreactor systems, e.g., the eZ control and pilot bioreactors (Applikon), Ralf and KLF (Bioengineering), BioFlo (Eppendorf), Labfors and Techfors (Infors HT), and D-DCU (Sartorius). It should be noted that the actual differences between connection types in benchtop and pilot scales are much less pronounced than the data in Table 2 would suggest.

No application could be found where a stuffing box is used as a sterile barrier in the investigated “off the shelf” bioreactors. The lip seal is preferred for simple and cost-effective operations, and its range of application can be clearly defined. This type of seal is used less frequently than mechanical seals and, in the data examined, is primarily found in top-driven glass bioreactors with less than 20 L working volumes, e.g., from Applikon, Belach Bioteknik, Broadley James, and Eppendorf. However, no such strict distinctions can be made for magnetic couplings and mechanical seals. These are used in single-use as well as glass and stainless steel systems, in top- and bottom-driven systems and for both microbial and cell culture applications (Table 3). Nevertheless, even if there are no strict rules, certain trends can be identified. Magnetic couplings are used in most single-use systems, which are mainly designed for cell culture processes with high sterility requirements. All types of seals are used for cell culture processes in reusable bioreactors, but double mechanical seals are suggested as a good alternative to magnetic coupling for sterile connections. Bottom agitators are more often equipped with magnetic couplings and top agitators with mechanical seals. It should be noted that stuffing boxes and lip seals are not used for bottom installation due to their poor hygienic suitability and tendency to leak. The dominance of top-driven systems, as mentioned by several authors, could not be confirmed in our study. For purely microbial bioreactors, mechanical and lip seals are dominant but not used exclusively, especially if a similar designed cell culture bioreactor is available from the same vendor. However, this overlapping use of mechanical and magnetic couplings does not necessarily indicate a misunderstanding of the merits of the individual approaches. Economic and technological reasons can play decisive roles, with targeted research increasing the area of application and reducing price. A simplified guideline for seal selection is depicted in Fig. 6.

Research and development are mainly being performed in the field of biopharmaceutical production processes using magnetically sealed bioreactors, where typical application volumes are 500 L for bacterial and 3000 L for mammalian cell cultures. The application of magnetically drives is limited by the viscosity of the culture broth (Matthews 2008) and the inability of the torque dependent power input to disperse gases and achieve homogeneity in the vessel (Stanbury et al. 2017). The maximum possible torque is defined by the magnetic field strength and is independent of the motor power, meaning the coupling breaks when the load limit is exceeded (Dickey 2015). Therefore, magnetic drives are mainly used at small scales and in single-use vessels (Table 3). Systems of up to 20 m^3^ with magnetic couplings (ZETA GmbH n.d.) and even 30 m^3^ (Dickey 2015) are especially common for applications in non-biological mixing processes or preparation systems that do not require fast mixing times or high mass transfer. Suppliers of such agitator systems include Alfa-Laval Mid Europe GmbH, MAVAG AG, liquitec AG, PRG GmbH, and ZETA GmbH. However, technical progress has also recently been made in terms of the use of magnetic couplings in larger bioprocesses. With their adapted magnetic system, Suleiko et al. (2020) demonstrated a drive with a torque of 200 Nm that is suitable for biological applications at a scale of 15 m^3^. ZETA’s magnetic bottom-mounted agitator for bioreactors with a torque of more than 400 Nm was also evaluated at the same scale (ZETA GmbH n.d.; Rosseburg et al. 2018; Fitschen et al. 2019), delivering results that predict applicability at scales up to 30 m^3^ (ZETA GmbH 2020a). Although their initial industrial use was controversial because of biological (hygienic design and SIP/CIP capability), mechanical, and chemical safety (possible abrasion in bearing-based systems) concerns, as well as the early lack of speed and torque monitoring (Eibl et al. 1996), magnetic agitators have been able to gain more and more acceptance, as a result of increased qualification and validation. Advantages such as hermetic separation between the product side and the environment, inclusion in measurement and control strategies, and the use of bottom-mounted magnetic agitators for especially low volumes of less than 10% of the maximum vessel volume, which make them suitable for multipurpose plants (Eibl and Schindler 2004), have also contributed to their increased acceptance. This has led to a situation in which magnetic couplings are not only used for pathogenic organisms, i.e., when containment is the main concern. Previously, the mechanical seal was the first choice for high-torque processes that required effective CIP procedures (Krahe 2003). However, this has changed with the advent of so-called floating bearings for slightly oscillating bottom-mounted magnetic agitators with a simple bearing journal and a sufficiently large gap between the impeller and containment shell. The resulting slight lifting and displacement effects ensure an axial flow with Taylor vortices in the gap, which support CIP and SIP strategies and prevent accumulation and subsequent contamination during the process (Eibl and Schindler 2004).

In the following sections, the four most typical seals are considered, starting with the stuffing box which is nowadays only used for non-sterile processes.

### Stuffing boxes

**Fig. 2:**
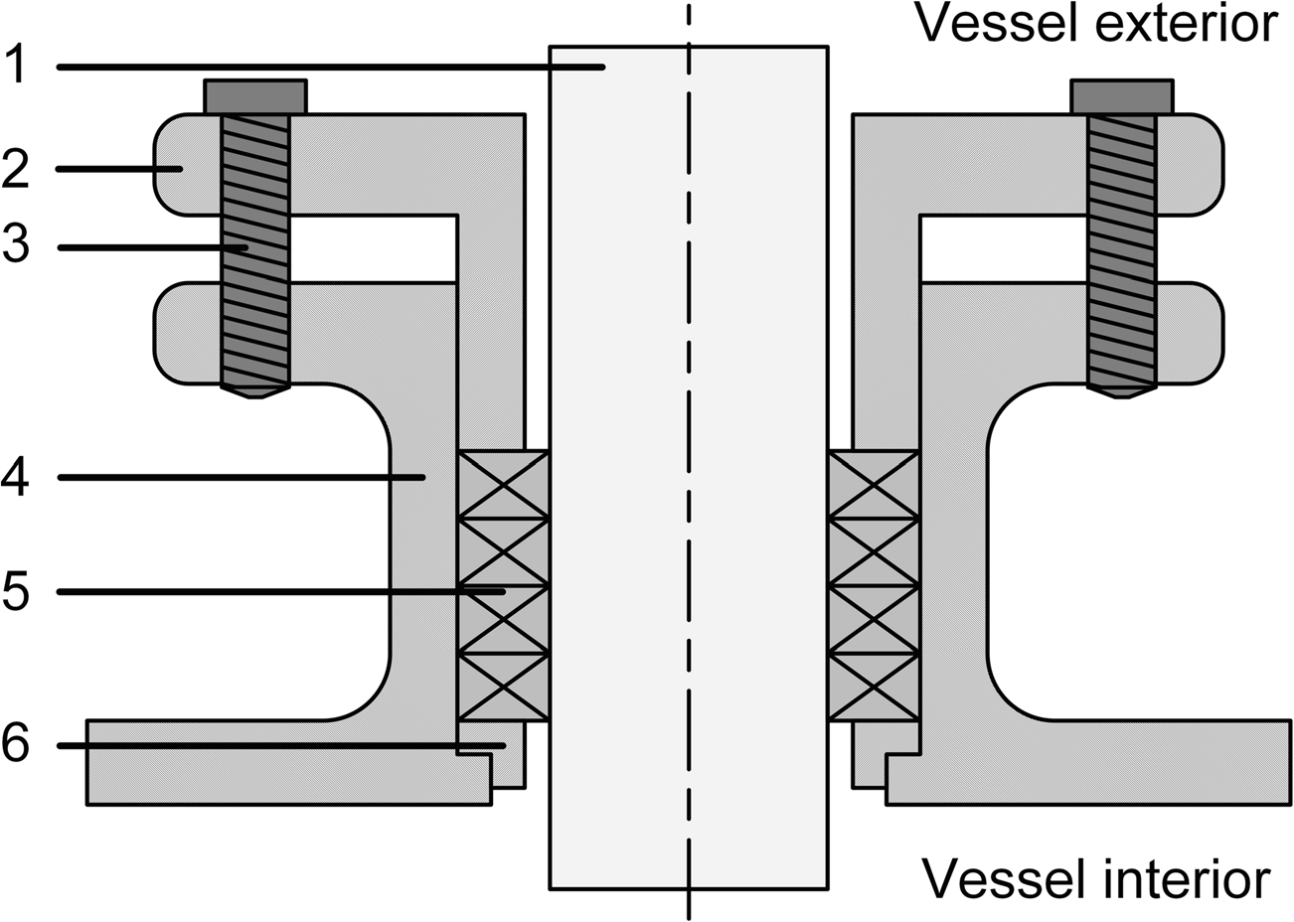
Stuffing box consisting of the shaft (1), gland (2), screws (3), housing (4), packing (5), and base bushing (6)

Stuffing boxes (Fig. 2) are one of the earliest dynamic sealing technologies and are used almost exclusively until the 1950s (Wilke et al. 1988; EKATO Holding GmbH 2012). In the early days of steamships, they were often used to seal the drive shaft as it passed through the hull, usually in the form of grease-soaked cloths stuffed between the stern tube and a housing. The basic structure of these versatile but very simple seals, which are primarily used for top-driven systems, consists of a casing around the shaft, which is fixed to the vessel and filled with a compression sealing material that minimizes leakages (Dickey and Fasano 2003). This packing material is why they are also known as packed gland seals. In the simplest case, this packing material can be hemp strings soaked in paraffin, which are stacked in the housing and compressed with the help of an adjustable plate and ring (gland and gland follower ring) that seal the gap between the shaft and the housing (Ignatowitz 1997). Nowadays, the packing tends to consist of one or more packing rings of different materials with different shapes (Mörl and Gelbe 2018). For instance, there are lamellar packing rings, which consist of corrugated metal inserts made of chromium steel, nickel, copper, or lead layered in cotton, asbestos (Ignatowitz 1997), acrylic, PTFE (polytetrafluoroethylene), Kevlar (aramid fibers), or graphite filaments (Dickey and Fasano 2003; Haberhauer 2014). Another possibility is foil packing rings, which consist of a fiber core wrapped in aluminum or other alloys (Dickey and Fasano 2003; Mörl and Gelbe 2018). Alternatively, there are also self-lubricating hollow rings made of lead or copper, which are filled with a graphite lubricant that can escape through small radial holes pointing towards the rotating shaft. In wedge sleeve packing rings, the axial tension on the wedge ring exerts pressure on the soft material insert, which is transferred to the running surface via a sleeve ring (Mörl and Gelbe 2018). Depending on the design and the packing material, stuffing boxes can be used for shaft diameters of 10–200 mm, temperatures of up to 520 °C, and pressures of up to 300 bar, but only low sliding speeds of approx. 0.3 m/s (Mörl and Gelbe 2018). Although sterile sealing cannot be achieved, a more hygienic seal can be achieved by using two stuffing boxes separated from each other by a steam-loaded flushing ring. Additional lubricants are injected into the stuffing boxes to ensure they remain gas-tight, even at high pressures (Menkel 1992).

Since the packing deforms plastically and wears out due to friction, the gland must be tightened from time to time and eventually replaced (Ignatowitz 1997). A problem that may be associated with this type of seal is product impurity resulting from packing material fibers, metal abrasion from the agitator shaft, and lubricants (Dickey and Fasano 2003). Since no statements can be made about the leakage rates through such seals, it is difficult to comply with environmental regulations and hygienic design concepts. Therefore, stuffing boxes are being used less frequently in both the chemical and pharmaceutical industries and are being replaced by mechanical seals (Wilke et al. 1988; Menkel 1992; EKATO Holding GmbH 2012). Fields of application where they are still used however are centrifugal pumps, compressors, and high-pressure axial-piston pumps as well as open cultivation systems (Haberhauer and Bodenstein 2014).

### Lip seal

**Fig. 3:**
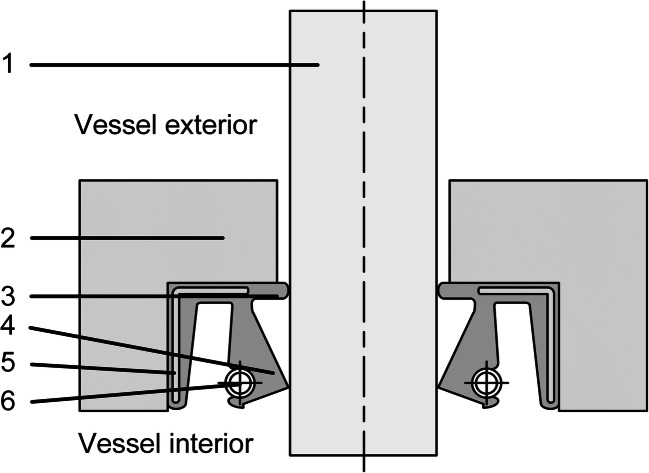
Radial shaft seal consisting of the shaft (1), housing (2), protective lip (3), sealing lip (4), metal stiffening ring (5), and tension spring ring (6)

Lip seals are probably the simplest and most cost-effective seals used in stirring technology (Dickey and Fasano 2003; Jagani et al. 2010). They are used for both axial and radial seals (EKATO Holding GmbH 2012), with radial shaft seals being the most common application (Haberhauer and Bodenstein 2014). Compared to other seal types, they deliver low sealing efficiency (Jagani et al. 2010) but can achieve a strong seal and have a long lifetime when used in small installations (Haberhauer and Bodenstein 2014). Radial shaft seals (Fig. 3) are usually used for gear shafts (Ignatowitz 1997) to seal lubricating grease and oil. Although they generally allow rotational speeds of up to 35 m/s, they often cannot withstand temperatures above 100 °C and pressures above 0.5 bar (Haberhauer 2014; Haberhauer and Bodenstein 2014). Nevertheless, they can be used in stirred vessels by implementing sophisticated shaft bearings that only allow shaft deflections of up to 0.01 mm, so that even higher pressures of up to 6 bar can be sealed (EKATO Holding GmbH 2012). Even if the seal is initially satisfactory, over time and particularly at high speeds, rapid wear does occur, which will cause the system to leak. Therefore, these seals are not suitable for long-term or continuous cultures in the pharmaceutical industry, since a permanent sterile barrier cannot be guaranteed (Jagani et al. 2010). They are therefore commonly used to keep dirt out of tanks at atmospheric pressure and to prevent the unhindered release of process vapors into the environment (Dickey and Fasano 2003).

The dynamic seal with the shaft is created by a sealing lip made of an elastomer or PTFE, with the required contact pressure in the radial direction being achieved by a tension spring ring. In addition, a rubber-like outer surface creates a static seal with the container (Ignatowitz 1997; Haberhauer 2014).

### Mechanical seal

**Fig. 4:**
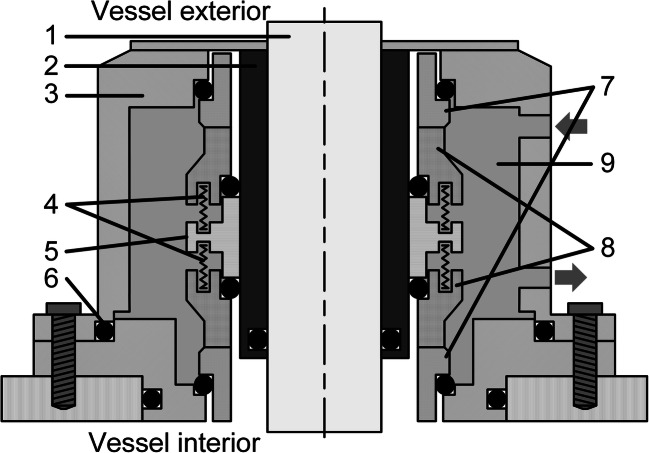
Double mechanical seal consisting of the shaft (1), bushing (2), housing (3), springs (4), spacer ring (5), O-rings (6), on the housing mounted static elements (7), on the shaft mounted dynamic elements (8), and flushing chamber with sealing liquid (9)

Mechanical seals, which are considered to be technically tight, have been an alternative to the previously mentioned sealing systems in agitator technology since the 1950s (EKATO Holding GmbH 2012), and they still meet today’s requirements for agitated cultivation systems in the biopharmaceutical industry (Menkel 1992; ASME 2019). The seal is formed by two sealing elements sliding axially against each other. One of these elements is dynamic because it is attached to the rotating agitator shaft. The dynamic element slides under axial compression on a statically mounted counter element that is located on the vessel. To ensure a permanent seal at a variety of temperatures at the same time as the sealing elements are being subjected to abrasion, the dynamic seal is spring-mounted in the axial direction so that the seal gap is kept tight. It is also necessary to create a liquid film between both elements in order to form a seal, since otherwise heat would be generated and excessive wear would occur (Menkel 1992; Dickey and Fasano 2003). This simple arrangement is called a single mechanical seal, which is usually lubricated by the medium present in the vessel (Matthews 2008). However, even these single mechanical seals pose a risk of contamination, leakage of the entire contents of the vessel in bottom-mounted systems, or aerosol formation in top-mounted systems. For these reasons, and especially for higher risk category organisms, double (Fig. 4) or even triple-acting mechanical seals are used. These consist of several pairs of single mechanical seals connected in series. In the case of a double mechanical seal, one pair of sealing elements seals the inside of the product chamber and a second pair the outside (Menkel 1992; Matthews 2008; Jagani et al. 2010; Hinrichs et al. 2018). The space between the two sealing pairs serves as a flushing chamber, which is filled with a sterile sealing liquid. This provides lubrication, cooling, and a discharge of abrasion while also preventing liquid from escaping from the vessel and stopping contaminants from entering from the atmospheric environment. A product-compatible sealing liquid is pressurized so that the sterile barrier is maintained, even in the event of a small leakage. This and sterilization of the intermediate space are carried out using pressurization systems, which in their simplest form use clean steam or clean steam condensate and compressed air (Chisti and Moo-Young 1994; Ignatowitz 1997; EKATO Holding GmbH 2012). A more detailed description of pressurization systems is provided by EKATO Holding GmbH (2012).

In addition to static and dynamic sealing elements, which are often made of silicon carbide, carbon graphite, or composites of both materials, static fluorocarbon, O-ring seals are also required to seal the contact surfaces between the vessel, the shaft, and the sealing elements (EKATO Holding GmbH 2012). This allows mechanical seals to be used for shaft diameters ranging from 5 to 500 mm at temperatures from −200 to +450 °C, pressures up to 450 bar, and rotational speeds of up to 100 m/s (Mörl and Gelbe 2018).

Although mechanical seals are the most commonly used seals in agitator systems, due to their superior durability and lower probability of contamination (Jagani et al. 2010), they are often the main cause of contamination problems. This is usually due to improper operation or failure to carry out proactive maintenance intervals in order to save costs (Junker et al. 2006).

### Hermetic seal—magnetic coupling

**Fig. 5:**
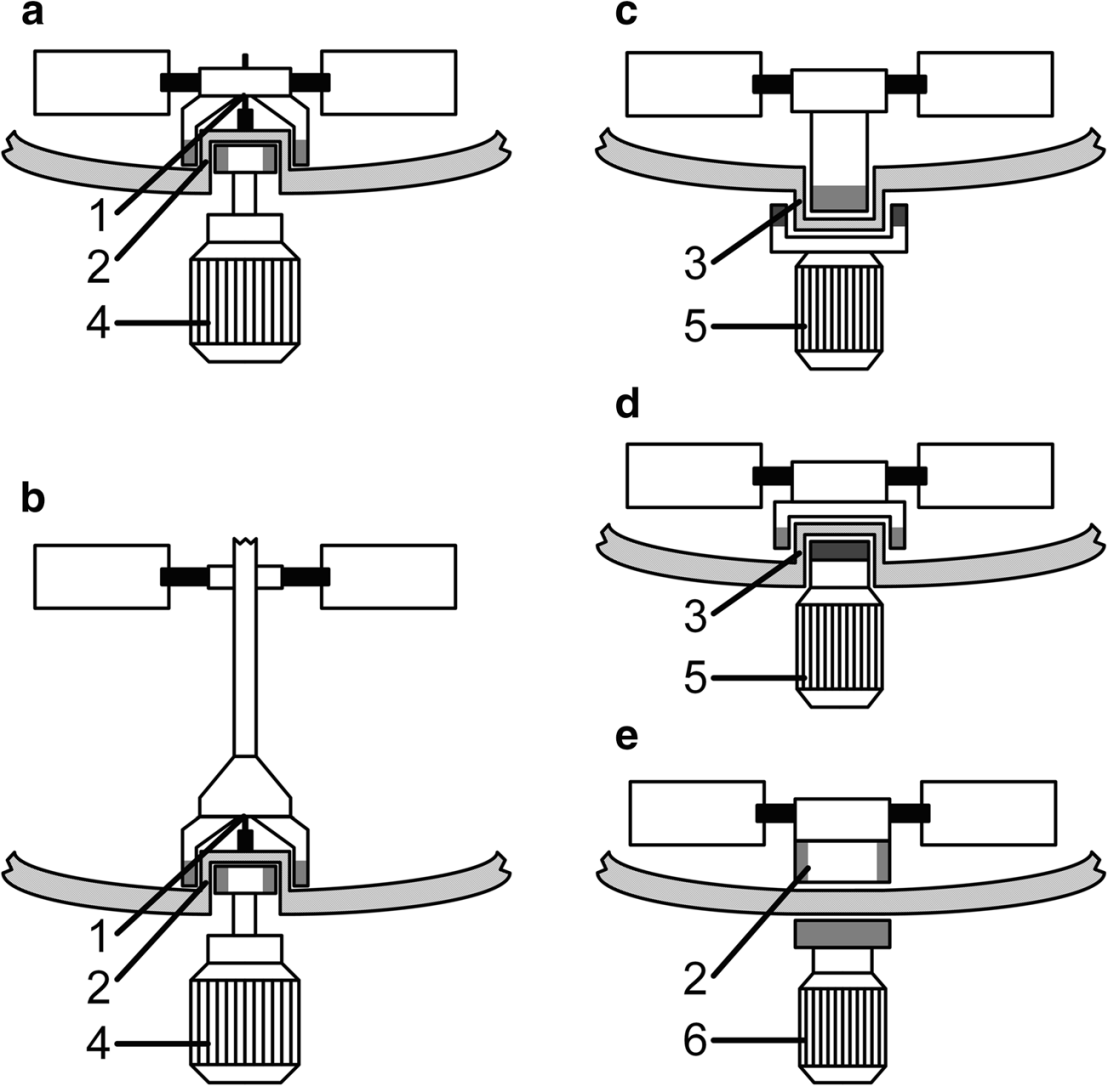
Hermetically sealed vessel with magnetic coupling of a bearing-mounted impeller (**a**), a bearing-mounted shaft with an impeller (**b**), a levitating impeller with interior rotor (**c**), a levitating impeller with exterior rotor (**d**), and a magnet-superconductor interaction-based impeller (**e**). The components involved are plain or roller bearings (1), passive (2) and active or passive magnets (3) for magnetic coupling, a drive (4), a stator and power electronics unit (5), and a drive using superconducting material (6)

In order to stir a hermetically sealed vessel, and thus reduce the risk of contamination to a minimum (Menkel 1992) and enable high-pressure processes at far more than 400 bar without the risk of leakage (Dickey 2015), the energy to the impeller must be supplied through the closed vessel wall (EKATO Holding GmbH 2012). For this purpose, power is transmitted using magnetic fields. Different impeller or stirrer shaft assemblies for magnetically agitated bioreactors are depicted in Fig. 5. This results in a coupling system that, unlike the systems described above, can be completely wear-free, thus guaranteeing longevity (Hinrichs et al. 2018). However, the rotational power that can be transmitted is limited by the maximum torque, which is itself limited by the strength of the magnets. Furthermore, these magnets often include rare earth materials (Dickey 2015). The field of application of such systems is defined by the temperature resistance of the magnets, which means that this type of coupling is mainly implemented for processes operating at moderate temperatures and sterilization processes at temperatures of up to 120 °C (Suleiko et al. 2020). This is due to the deterioration of ferromagnetic properties at high temperatures. These problems can be resolved by using very strong magnets made of neodymium in combination with transition metals to provide resistance to temperatures up to 300 °C (Weir et al. 2020). In industry, both bearing-based and levitated magnetic drive systems (as described below) are used.

#### Bearing-based systems

In bearing-based systems, the impeller is set in motion using permanent magnets located on the drive and the stirrer shaft, which are separated from each other by the containment shell. Since the bearing for the stirrer shaft and/or impeller is located inside the vessel, CIP-compatible ceramic plain and roller bearings made of zirconium oxide (EKATO Holding GmbH 2012) are often used for hygienic reasons (Hinrichs et al. 2018). The drive shaft outside the vessel is powered by a traditional motor element (Dickey 2015), which in some cases can require a large construction on top of or below the vessel (Sun et al. 2013). A further disadvantage is that if there is insufficient lubrication of the bearing, friction will occur, which may result in attrition of the material and impact product purity (Reichert et al. 2012; Haberhauer 2014). The system is generally lubricated by the medium or culture broth; therefore, running the system dry should be avoided. Particularly in bottom-driven systems, in which single bearing journals are located directly in the medium (Dickey 2015), care must be taken to ensure there is a sufficiently large gap between the containment shell and the directly mounted impeller in order to avoid highly damaging shear rates (ZETA GmbH 2020b) and to enable CIP and SIP procedures to be easily performed. Should hydrodynamic flow effects not provide sufficient cleaning in the gap between the containment shell and the impeller, plain bearings with additional radial and axial grooves on the sliding surfaces to achieve optimal cleaning are also available (Hinrichs et al. 2018; ASME 2019). In contrast, top-driven systems are either also supported by ceramic roller bearings or are equipped with commercial oil-lubricated roller bearings inside a bearing chamber, separated from the vessel interior by a mechanical seal (EKATO Holding GmbH 2012). These systems are available for vessels up to 400 L with torque ratings from 0.7 to 115 Nm, while bottom-mounted systems are available for applications up to 150 Nm in containment vessels of up to 30 m^3^ (Dickey 2015).

#### Levitated systems

A basic distinction can be made between two different types of levitation drive technology, in which the impeller is magnetically supported inside the vessel.

Superconducting mixers use non-contact magnetic coupling between conventional permanent magnets in the impeller and a superconducting material in the drive below the vessel (Koyama et al. 2006). The superconducting material detects the magnetic field generated by the permanent magnets, stores it and attempts to fix it in a position of equilibrium to keep the magnets and the impeller in position when external forces are applied. The very stable coupling resulting from the magnet-superconductor interaction allows speeds of up to 210 rpm and a temperature range of 4–60 °C. Since the drive unit is mobile, it can be used successively with several vessels up to fluid volumes of 1000 L (Sartorius Stedim Biotech GmbH 2013; Pall Corporation 2016).

A different approach is usually applied in bearingless pump systems, which are used in biotechnology (Schöb 2002) as well as in medicine as left ventricular assist devices for the treatment of heart disorders (Schöb and Loree 2008) or to maintain blood circulation during heart transplants (Sung et al. 2015). Bearingless pump systems are characterized by low shear stresses (Blaschczok et al. 2013; Dittler et al. 2014; Schirmer and Eibl 2018), which has led to their use in the operation of bioreactors (Reichert et al. 2009; Schirmer et al. 2018) and mixing systems in the pharmaceutical industry (Sartorius Stedim Biotech GmbH 2019).

A brushless drive and the necessary magnetic bearing are accommodated in a single unit, meaning a shaft and mechanical bearing for driving the rotor are not required. As a result, maintenance and service costs are reduced, since there are no wearing parts and lubrication is not required (Reichert et al. 2012). Using electromagnets makes the magnetic bearing active, while using permanent magnets results in a passive magnetic bearing. To stabilize the impeller, rotation and translation along the three axes of motion must all be controlled. However, only five of the total of six degrees of freedom require stabilization, since the rotation of the rotor along the main axis is determined by the drive. Stabilization of the remaining five degrees of freedom is achieved by a combination of passive and active magnetic bearings. Passive magnetic bearings use permanent magnets made of rare earth elements, which are characterized by their high-energy density and small space requirements. In contrast, active magnetic bearings are used when precise position control or bearing rigidity is required (Nussbaumer et al. 2011).

The simplest design for a bearingless magnetic motor is the bearingless slice motor, which was first developed by Barletta (1998). In this type of drive, the rotor consists of a ring-shaped, two-pole, permanent magnet that is magnetized diametrically to the rotor plane (Bösch 2004). Three of the six degrees of freedom are stabilized either passively or actively. A permanent magnet ring can be used to stabilize both rotations along the *x*- and *z*-axes and translation along the *y*-axis. Centering the rotor on the *x*- and *z*-axis origin is achieved by two active magnetic bearings, and the last degree of freedom, rotation around the *y*-axis, is actively controlled by the motor drive (Schöb and Loree 2008; Nussbaumer et al. 2011). To stabilize the radial position of the rotor at high speeds, the magnetic field is adjusted 10,000 times per second (Levitronix GmbH 2020).

Levitation-driven impellers create no friction or mechanical stress during the mixing process, meaning no particles are generated that could contaminate the product. Therefore, levitated systems are suitable for ultrapure and sterile mixing processes (Schöb 2002; Sartorius Stedim Biotech GmbH 2013).

### Decision tree for seal type selection

**Fig. 6:**
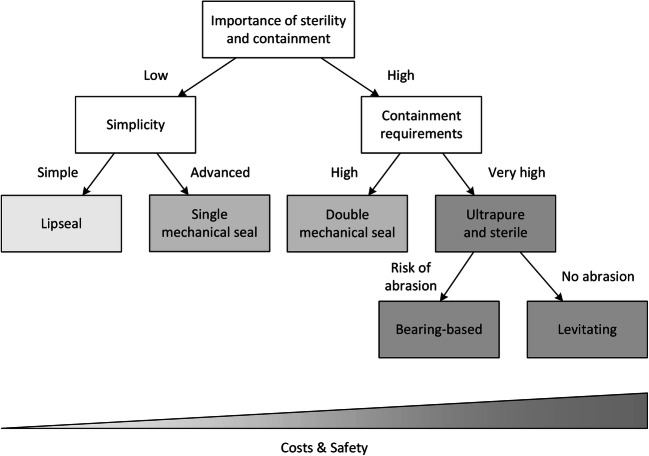
Sealing selection: A decision tree based on bioprocess demands and economic considerations. Cheaper sealing approaches may be replaced by ones that create a tighter seal (e.g., lip seal by mechanical seal)

This section provides recommendations for the selection of a suitable seal based on the expression organism and process mode. The selection is based on a compromise between sterility requirements and economic considerations. In most cases, a higher quality seal can of course be used, provided that the process conditions do not prohibit this (e.g., high speeds for microbial processes can be achieved more easily with a mechanical seal than with a magnetic coupling). A simple procedure for selecting the correct seal for a given purpose is shown in Fig. 6. The classification is primarily based on sterility requirements and secondarily on economic or containment considerations. Open microbial and algae-based processes as well as short experiments in single-use systems can therefore be carried out with simple mechanical seals. Cell culture processes and processes in which the escape of organisms or their products into the environment must be avoided at all costs should be carried out with multiple mechanical seals or magnetic couplings. A more precise matching of seal types to individual organism types and process modes, also taking into account the products to be obtained, is given in Table 4. It should be noted that neither the decision tree nor Table 4 are rigid rules and many processes can also be performed with other seals, but this assignment allows a safe and at the same time inexpensive selection to be made.

**Table 4:** Sealing recommendations based on organism, product and process mode

Organism (example)	Target	Batch	Fed-batch	Continuous/perfusion
Bacteria	Biofuels, small molecules	Stuffing box/lip seal	Lip seal	Mechanical seal
	Biopharmaceuticals	Mechanical seal	Mechanical seal	Mechanical seal
Yeast	Ethanol fermentation	Lip seal	Lip seal	Mechanical seal
	Protein production	Mechanical seal	Mechanical seal	Mechanical seal
Algae	Phototrophic biomass production	Stuffing box/lip seal	Lip seal	Mechanical seal
	Heterotrophic product synthesis	Mechanical seal	Mechanical seal	Mechanical seal
Plant	Secondary metabolites	Mechanical seal	Mechanical seal	Mechanical seal
	Recombinant proteins	Mechanical seal/magnetic coupling	Mechanical seal/magnetic coupling	Mechanical seal/magnetic coupling
	Cellular agriculture	Mechanical seal	Mechanical seal	Mechanical seal
Insect	BEVS, biopesticides	Mechanical seal/magnetic coupling	Mechanical seal/magnetic coupling	Magnetic coupling*
Mammalian	Recombinant proteins	Mechanical seal/magnetic coupling*	Magnetic coupling*	Magnetic coupling*
Stem cells	Regenerative medicine	Magnetic coupling*	Magnetic coupling*	Magnetic coupling*

*BEVS* Baculorvirus protein expression vector system

*For these processes, the authors recommend the use of single-use systems

## Conclusions and perspectives

The use and selection of seals or motor couplings are of utmost importance for ensuring sterility in biotechnological production processes. The most commonly used seals at laboratory and pilot scales are the lip seal, the mechanical seal, and the magnetic coupling. The lip seal is the simplest of the seal types and is mainly used in smaller top-driven systems. Mechanical seals and magnetic couplings are used for microbial and cell cultures in single-use or reusable bioreactors systems with top or bottom drives, depending on the manufacturer. The advantages of either system—better sterility of magnetic coupling and higher possible torques with mechanical seals—do not seem to significantly influence manufacturers’ seal choices, particularly at smaller scales. Increasing demands on sterility and process safety favor magnetically coupled systems, which are the subject of increased research. Therefore, the basic torque limitation problem of magnetically driven systems should be minimized by progressing technologies and using neodymium magnets (Suleiko et al. 2020), which are the strongest permanent magnets currently available on the market. The growing trend in recent years to use magnetically driven systems in more complex mixing processes, such as in microbial and cell culture applications (ZETA GmbH n.d.), has also accelerated development up to current scales of 30–40 m^3^ (MAVAG AG 2020; ZETA GmbH 2020a). Similarly, increased use of levitating bottom-mounted agitator systems, which are almost all based on the pump drive technology commercialized by Levitronix AG, can also be observed. Due to the wide performance range, these systems are suitable for both powerful and low shear mixing applications. This flexibility has also been demonstrated in a 2 L bioreactor system using Levitronix pump drives (BPS-i30 and BPS-i100) (Schirmer et al. 2018).

## Data Availability

Not applicable

## References

[CR1] Abecasis B, Aguiar T, Arnault É, Costa R, Gomes-Alves P, Aspegren A, Serra M, Alves PM (2017). Expansion of 3D human induced pluripotent stem cell aggregates in bioreactors: bioprocess intensification and scaling-up approaches. J Biotechnol.

[CR2] Adler I, Fiechter A (1983). Charakterisierung von Bioreaktoren mit biologischen Testsystemen. Chem Ing Tech.

[CR3] Akhnoukh R, Kretzmer G, Schügerl K (1996). On-line monitoring and control of the cultivation of *Spodoptera frugiperda Sf*9 insect cells and β-galactosidase production by *Autographa californica* virus vector. Enzym Microb Technol.

[CR4] Arshad M, Hussain T, Iqbal M, Abbas M (2017). Enhanced ethanol production at commercial scale from molasses using high gravity technology by mutant *S. cerevisiae*. Braz J Microbiol.

[CR5] ASME (2019). Bioprocessing equipment (BPE).

[CR6] Assirelli M, Bujalski W, Eaglesham A, Nienow AW (2008). Macro- and micromixing studies in an unbaffled vessel agitated by a Rushton turbine. Chem Eng Sci.

[CR7] Barletta N (1998) Der lagerlose Scheibenmotor. Dissertation, ETH Zürich

[CR8] Bausch M, Schultheiss C, Sieck JB (2019). Recommendations for comparison of productivity between fed-batch and perfusion processes. Biotechnol J.

[CR9] Benvenuti G, Bosma R, Ji F, Lamers P, Barbosa MJ, Wijffels RH (2016). Batch and semi-continuous microalgal TAG production in lab-scale and outdoor photobioreactors. J Appl Phycol.

[CR10] Birch JR, Flickinger MC (2010). Suspension Culture, Animal Cells. Encyclopedia of Industrial Biotechnology.

[CR11] Blaschczok K, Kaiser SC, Löffelholz C, Imseng N, Burkart J, Bösch P, Dornfeld W, Eibl R, Eibl D (2013). Investigations on mechanical stress caused to CHO suspension cells by standard and single-use pumps. Chem Ing Tech.

[CR12] Bösch PN (2004) Lagerlose Scheibenläufermotoren höherer Leistung. Dissertation, ETH Zürich

[CR13] Bruder S, Reifenrath M, Thomik T, Boles E, Herzog K (2016). Parallelised online biomass monitoring in shake flasks enables efficient strain and carbon source dependent growth characterisation of *Saccharomyces cerevisiae*. Microb Cell Factories.

[CR14] Brunner M, Fricke J, Kroll P, Herwig C (2017). Investigation of the interactions of critical scale-up parameters (pH, pO2 and pCO2) on CHO batch performance and critical quality attributes. Bioprocess Biosyst Eng.

[CR15] Buffo MM, Corrêa LJ, Esperança MN, Cruz AJG, Farinas CS, Badino AC (2016). Influence of dual-impeller type and configuration on oxygen transfer, power consumption, and shear rate in a stirred tank bioreactor. Biochem Eng J.

[CR16] Catapano G, Czermak P, Eibl R, Eibl D, Pörtner R, Eibl R, Eibl D, Pörtner R, Catapano G, Czermak P (2009). Bioreactor design and scale-up. Cell and Tissue Reaction Engineering.

[CR17] Chisti Y, Ratledge C, Kristiansen B (2006). Bioreactor design. Basic Biotechnology.

[CR18] Chisti Y, Soetaert W, Vandamme EJ (2010). Fermentation Technology. Industrial biotechnology.

[CR19] Chisti Y, Moo-Young M (1994). Clean-in-place systems for industrial bioreactors: design, validation and operation. J Ind Microbiol.

[CR20] Chmiel H, Weuster-Botz D, Chmiel H, Takors R, Weuster-Botz D (2018). Bioreaktoren. Bioprozesstechnik.

[CR21] Clapp KP, Castan A, Lindskog EK, Jagschies G, Lindskog E, Lacki K, Galliher PM (2018). Upstream processing equipment. Biopharmaceutical Processing.

[CR22] Creathorn A (2003) Design considerations for a large mixer used in an agitated column application. Proc Twent Int Pump Users Symp 83–90. 10.21423/R15H4H

[CR23] DECHEMA (1982). Arbeitsmethoden für die Biotechnologie : Referenz-Bioreaktoren, Vergleichstests für Fermentationen, sichere Biotechnologie.

[CR24] DECHEMA (1991). Standardisierungs- und Ausrüstungsempfehlungen für Bioreaktoren und periphere Einrichtungen.

[CR25] Dickey DS, Kresta SM, Etchells AW, Dickey DS, Atiemo-Obeng VA, Forum NAM (2015). Magnetic Drives for Mixers. Advances in industrial mixing: a companion to the Handbook of industrial mixing.

[CR26] Dickey DS, Fasano JB, Paul EL, Atiemo-Obeng VA, Kresta SM (2003). Mechanical Design of Mixing Equipment. Handbook of Industrial Mixing.

[CR27] Dittler I, Kaiser SC, Blaschczok K, Löffelholz C, Bösch P, Dornfeld W, Schöb R, Rojahn J, Kraume M, Eibl D (2014). A cost-effective and reliable method to predict mechanical stress in single-use and standard pumps. Eng Life Sci.

[CR28] Eibl D, Eibl R, Eibl R, Eibl D (2019). Single-use equipment in biopharmaceutical manufacture. Single-Use Technology in Biopharmaceutical Manufacture.

[CR29] Eibl D, Schindler H (2004) Magnetrührwerke in der Biotechnologie. In: 4. Köthener Rührer-Kolloqium. Hochschule Anhalt (FH), Köthen, pp 102–114

[CR30] Eibl D, Jenny D, Meier HP (1996). Einsatz eines Magnetrührwerkes in der Lebensmittel- und Pharmaindustrie. BioWorld.

[CR31] Eibl R, Jossen V, Eibl D (2018). Einweg-Bioreaktoren. Chem Unserer Zeit.

[CR32] EKATO Holding GmbH (2012) Ekato. The Book. EKATO Holding GmbH, Freiburg

[CR33] Elias CB, Zeiser A, Bédard C, Kamen AA (2000) Enhanced growth of *sf*-9 cells to a maximum density of 5.2 × 10^7^ cells per mL and production of β-galactosidase at high cell density by fed batch culture. Biotechnol Bioeng 68:381–388. 10.1002/(sici)1097-0290(20000520)68:4<381::aid-bit3>3.0.co;2-d 10745206

[CR34] Fitschen J, Maly M, Rosseburg A, Wutz J, Wucherpfennig T, Schlüter M (2019). Influence of spacing of multiple impellers on power input in an industrial-scale aerated stirred tank reactor. Chem Ing Tech.

[CR35] Garcia-Ochoa F, Gomez E (2009). Bioreactor scale-up and oxygen transfer rate in microbial processes: An overview. Biotechnol Adv.

[CR36] Gleich D, Weyl R (eds) (2006) Abschlusselemente. In: Apparateelemente. Springer-Verlag, Berlin/Heidelberg, pp 117–190

[CR37] Haberhauer H, Skolaut W (2014). Dichtungen - die Funktion von Maschinenelementen gewährleisten. Maschinenbau.

[CR38] Haberhauer H, Bodenstein F (eds) (2014) Dichtungen. In: Maschinenelemente. Springer Berlin Heidelberg, Berlin/Heidelberg, pp 259–282

[CR39] Haigh J, Schmidt SR, Vicalvi J, Winterhalter C (2020). 17th Annual Report and Survey on Biopharmaceutical Manufacturing Capacity and Production.

[CR40] Hausjell J, Weissensteiner J, Molitor C, Halbwirth H, Spadiut O (2018). *E. coli* HMS174(DE3) is a sustainable alternative to BL21(DE3). Microb Cell Factories.

[CR41] Hemrajani RR, Tatterson GB, Paul EL, Atiemo-Obeng VA, Kresta SM (2003). Mechanically Stirred Vessels. Handbook of Industrial Mixing.

[CR42] Hinrichs J, Buck H, Hauser G, Chmiel H, Takors R, Weuster-Botz D (2018). Sterilisation und Sterildesign. Bioprozesstechnik.

[CR43] Holland T, Blessing D, Hellwig S, Sack M (2013). The in-line measurement of plant cell biomass using radio frequency impedance spectroscopy as a component of process analytical technology. Biotechnol J.

[CR44] Ignatowitz E (1997). Chemietechnik.

[CR45] Imseng N, Steiger N, Frasson D, Sievers M, Tappe A, Greller G, Eibl D, Eibl R (2014). Single-use wave-mixed versus stirred bioreactors for insect-cell/BEVS-based protein expression at benchtop scale. Eng Life Sci.

[CR46] Jagani HV, Hebbar K, Gang SS, Palanimuthu VR, Hariharapura RC, Rao JV (2010). An Overview of fermenter and the design considerations to enhance its productivity. Pharmacologyonline.

[CR47] Jardin BA, Montes J, Lanthier S, Tran R, Elias C (2007). High cell density fed batch and perfusion processes for stable non-viral expression of secreted alkaline phosphatase (SEAP) using insect cells: Comparison to a batch *Sf*-9-BEV system. Biotechnol Bioeng.

[CR48] Jossen V, Pörtner R, Kaiser SC, Kraume M, Eibl D, Eibl R, Eberli D (2014). Mass production of mesenchymal stem cells — impact of bioreactor design and flow conditions on proliferation and differentiation. Cells and Biomaterials in Regenerative Medicine.

[CR49] Jossen V, Schirmer C, Mostafa Sindi D, Eibl R, Kraume M, Pörtner R, Eibl D (2016). Theoretical and practical issues that are relevant when scaling up hmsc microcarrier production processes. Stem Cells Int.

[CR50] Jossen V, Eibl R, Pörtner R, Kraume M, Eibl D, Larroche C, Angeles Sanroman M, Du G, Ashok P (2017). Stirred bioreactors. Current developments in biotechnology and bioengineering.

[CR51] Jossen V, Eibl R, Eibl D, Eibl R, Eibl D (2019). Single-use bioreactors – an overview. Single-Use Technology in Biopharmaceutical Manufacture.

[CR52] Junker BH (2004). Scale-up methodologies for *Escherichia coli* and yeast fermentation processes. J Biosci Bioeng.

[CR53] Junker B, Lester M, Leporati J, Schmitt J, Kovatch M, Borysewicz S, Maciejak W, Seeley A, Hesse M, Connors N, Brix T, Creveling E, Salmon P (2006). Sustainable reduction of bioreactor contamination in an industrial fermentation pilot plant. J Biosci Bioeng.

[CR54] Kante RK, Vemula S, Somavarapu S, Mallu MR, Boje Gowd BH, Ronda SR (2018). Optimized upstream and downstream process conditions for the improved production of recombinant human asparaginase (rhASP) from *Escherichia coli* and its characterization. Biologicals.

[CR55] Korz DJ, Rinas U, Hellmuth K, Sanders EA, Deckwer W-D (1995). Simple fed-batch technique for high cell density cultivation of *Escherichia coli*. J Biotechnol.

[CR56] Koyama F, Akiyama S, Murakami M (2006). Developments of superconducting mixers for medical applications. Supercond Sci Technol.

[CR57] Krahe M (2003). Biochemical engineering. Ullmann’s Encyclopedia of Industrial Chemistry.

[CR58] Kumaresan T, Joshi JB (2006). Effect of impeller design on the flow pattern and mixing in stirred tanks. Chem Eng J.

[CR59] Lee S-Y, Kim D-I (2006). Perfusion cultivation of transgenic *Nicotiana tabacum* suspensions in bioreactor for recombinant protein production. J Microbiol Biotechnol.

[CR60] Lee S-Y, Kim YH, Roh YS, Myoung HJ, Lee KY, Kim D-I (2004) Bioreactor operation for transgenic *Nicotiana tabacum* cell cultures and continuous production of recombinant human granulocyte-macrophage colony-stimulating factor by perfusion culture. Enzym Microb Technol 35:663–671. 10.1016/j.enzmictec.2004.08.019

[CR61] Levitronix GmbH (2020) Bearingless Motor Technology. https://www.levitronix.com/en/bearingless-motors.html. Accessed 9 Aug 2020

[CR62] Li T, Bin CX, Chen JC, Wu Q, Chen GQ (2014). Open and continuous fermentation: Products, conditions and bioprocess economy. Biotechnol J.

[CR63] Liepe F, Sperling R, Jembere S (1998). Rührwerke: Theoretische Grundlagen, Auslegung und Bewertung.

[CR64] Liu J, Sun Z, Chen F, Pandey A, Lee D-J, Chisti Y, Soccol CR (2014). Heterotrophic Production of Algal Oils. Biofuels from Algae.

[CR65] Matthews G, McNeil B, Harvey LM (2008). Fermentation equipment selection: laboratory scale bioreactor design considerations. Practical Fermentation Technology.

[CR66] MAVAG AG (2020) MAVADRIVE®. http://mavag.com.gutenberg.ch-meta.net/_wys_files/MAVADRIVEBroschuere.pdf. Accessed 12 Nov 2020

[CR67] Menkel F (1992). Einführung in die Technik von Bioreaktoren.

[CR68] Meusel W, Löffelholz C, Husemann U, Dreher T, Greller G, Kauling J, Eibl D, Kleebank S, Bauer I, Glöckler R, Huber P, Kuhlmann W, John GT, Werner S, Kasier SC, Pörtner R, Kraume M (2016). Recommendations for process engineering characterisation of single-use bioreactors and mixing systems by using experimental methods.

[CR69] Meyer H-P, Schmidhalter DR (eds) (2014) The history and economic relevance of industrial scale suspension culture of living cells. In: Industrial scale suspension culture of living cells. Wiley-VCH Verlag GmbH & Co. KGaA, Weinheim, pp 1–38

[CR70] Meyer H-P, Minas W, Schmidhalter D, Wittmann C, Liao JC (2016). Industrial-scale fermentation. Industrial Biotechnology.

[CR71] Mirro R, Voll K (2009). Which impeller is right for your cell line?. Bioprocess Int.

[CR72] Mohd Azhar SH, Abdulla R, Jambo SA, Marbawi H, Gansau JA, Mohd Faik AA, Rodrigues KF (2017). Yeasts in sustainable bioethanol production: A review. Biochem Biophys Rep.

[CR73] Möller J, Hernández Rodríguez T, Müller J, Arndt L, Kuchemüller KB, Frahm B, Eibl R, Eibl D, Pörtner R (2020). Model uncertainty-based evaluation of process strategies during scale-up of biopharmaceutical processes. Comput Chem Eng.

[CR74] Mörl L, Gelbe H, Grote K-H, Bender B, Göhlich D (2018). Konstruktionselemente von Apparaten und Rohrleitungen. Dubbel.

[CR75] Najafpour GD (ed) (2015) Bioreactor design. In: Biochemical engineering and biotechnology, 2nd edn. Elsevier, Amsterdam, pp 193–226

[CR76] Nienow AW (1998). Hydrodynamics of stirred bioreactors. Appl Mech Rev.

[CR77] Nienow AW, Flickinger MC (2010). Impeller Selection for Animal Cell Culture. Encyclopedia of Industrial Biotechnology.

[CR78] Nienow AW, Isailovic B, Barrett TA (2016). Design and Performace of Single-Use, Stirred-Tank Bioreactors. Bioprocess Int.

[CR79] Nussbaumer T, Karutz P, Zurcher F, Kolar JW (2011). Magnetically levitated slice motors - an overview. IEEE Trans Ind Appl.

[CR80] Pahl M, Kraume M (2002). Mischtechnik, Aufgaben und Bedeutung. Mischen und Rühren.

[CR81] Pall Corporation (2016) LevMixer® System. https://shop.pall.com/INTERSHOP/web/WFS/PALL-PALLUS-Site/en_US/-/USD/ViewProductAttachment-OpenFile?LocaleId=&DirectoryPath=pdfs%2FBiopharmaceuticals&FileName=16-6543_USD2952b_LevMixer_SS.pdf&UnitName=PALL. Accessed 9 Aug 2020

[CR82] Penicot P, Muhr H, Plasari E, Villermaux J (1998) Influence of the Internal Crystallizer Geometry and the Operational Conditions on the Solid Product Quality. Chem Eng Technol 21:507–514.

[CR83] Raj AE, Karanth N, Pometto A, Shetty K, Paliyath G, Levin RE (2005). Fermentation technology and bioreactor design. Food Biotechnology.

[CR84] Reichert T, Nussbaumer T, Gruber W, Kolar JW (2009) Design of a novel bearingless permanent magnet motor for bioreactor applications. In: 2009 35th Annual Conference of IEEE Industrial Electronics. IEEE, pp 1086–1091

[CR85] Reichert T, Nussbaumer T, Kolar JW (2012). Bearingless 300 W PMSM for Bioreactor Mixing. IEEE Trans Ind Electron.

[CR86] Rosseburg A, Fitschen J, Wutz J, Wucherpfennig T, Schlüter M (2018). Hydrodynamic inhomogeneities in large scale stirred tanks – Influence on mixing time. Chem Eng Sci.

[CR87] Sartorius Stedim Biotech GmbH (2013) Standard Flexel® for LevMixer® 50 L to 1,000 L. http://sartorius-sd.com.ua/files/Mixing_system/Data_Flexel3D_LevMix_System_for_Palletank_50-1000l.pdf. Accessed 9 Aug 2020

[CR88] Sartorius Stedim Biotech GmbH (2019) Flexsafe® Pro Mixer Pre-designed Solutions. https://www.sartorius.com/download/343654/6/broch-flexsafe-promixer-pds-2548196-000-e-data.pdf. Accessed 9 Aug 2020

[CR89] Scargiali F, Tamburini A, Caputo G, Micale G (2017). On the assessment of power consumption and critical impeller speed in vortexing unbaffled stirred tanks. Chem Eng Res Des.

[CR90] Scheiblauer J, Scheiner S, Joksch M, Kavsek B (2018). Fermentation of *Saccharomyces cerevisiae* – Combining kinetic modeling and optimization techniques points out avenues to effective process design. J Theor Biol.

[CR91] Schiel O, Jarchow-Redecker K, Piehl G-W, Lehmann J, Berlin J (1984). Increased formation of cinnamoyl putrescines by fedbatch fermentation of cell suspension cultures of *Nicotiana tabacum*. Plant Cell Rep.

[CR92] Schirmer C, Eibl D (2018) Shear stress investigations of the magnetically levitated single-use centrifugal pump PuraLev® 600SU using the protein shear stress model for lysozyme. https://www.levitronix.com/en/technical-papers.html?file=files/dl__documents/Technical%20Papers%20Life%20Science/Shear%20stress%20investigations%20of%20the%20magnetically%20levitated%20single-use%20centrifugal%20pump%20PURALEV%20600SU%20using%20the%20protein%20shear%20stress%20model%20for%20lysozyme.pdf. Accessed 9 Aug 2020

[CR93] Schirmer C, Blaschczok K, Husemann U, Leupold M, Zahnow C, Rupprecht J, Glöckler R, Greller G, Pörtner R, Eibl R, Eibl D (2017). Standardized qualification of stirred bioreactors for microbial biopharmaceutical production processes. Chem Ing Tech.

[CR94] Schirmer C, Nussbaumer T, Schöb R, Pörtner R, Eibl R, Eibl D (2018) Development, Engineering and Biological Characterization of Stirred Tank Bioreactors. In: Yeh M-K, Chen Y-C (eds) Biopharmaceuticals. InTech, pp 87–108

[CR95] Schirmer C, Dreher T, Leupold M, Glaser R, Castan A, Brown J, Eibl D, Glöckler R (2019). Recommendation for biological evaluation of bioreactor performance for microbial processes.

[CR96] Schöb R (2002). Centrifugal pump without bearings or seals. World Pumps.

[CR97] Schöb R, Loree HM, Wintermantel E, Ha S-W (2008). Technische Systeme für den Herzersatz und die Herzunterstützung. Medizintechnik Life Science Engineering.

[CR98] Simaõ D, Arez F, Terasso AP, Pinto C, Sousa MFQ, Brito C, Alves PM (2016). Perfusion stirred-tank bioreactors for 3D differentiation of human neural stem cells. Methods Mol Biol.

[CR99] Stanbury PF, Whitaker A, Hall SJ (eds) (2017) Aeration and agitation. In: Principles of Fermentation Technology. Elsevier, pp 537–618

[CR100] Suleiko A, Vanags J, Konuhova M, Dubencovs K, Grigs O (2020). The application of novel magnetically coupled mixer drives in bioreactors of up to 15 m^3^. Biochem Eng J.

[CR101] Sun X, Chen L, Yang Z (2013). Overview of bearingless permanent-magnet synchronous motors. IEEE Trans Ind Electron.

[CR102] Sun H, Ren Y, Lao Y, Li X, Chen F (2020). A novel fed-batch strategy enhances lipid and astaxanthin productivity without compromising biomass of *Chromochloris zofingiensis*. Bioresour Technol.

[CR103] Sung S-Y, Hsu P-S, Chen J-L, Tsai C-S, Tsai Y-T, Lin C-Y, Lee C-Y, Ke H-Y, Lin Y-C (2015). Prolonged use of levitronix left ventricular assist device as a bridge to heart transplantation. Acta Cardiol Sin.

[CR104] Terpe K (2006). Overview of bacterial expression systems for heterologous protein production: from molecular and biochemical fundamentals to commercial systems. Appl Microbiol Biotechnol.

[CR105] Thermo Fisher Scientific Inc. (2019) HyPerforma DynaDrive single-use bioreactor (S.U.B.). https://assets.thermofisher.com/TFS-Assets/BPD/brochures/dynadrive-sub-brochure.pdf. Accessed 29 Sep 2020

[CR106] Tosa T, Sato T, Mori T, Chibata I (1974). Basic studies for continuous production of l-aspartic acid by immobilized *Escherichia coli* cells. Appl Microbiol.

[CR107] Travieso Córdoba L, Domínguez Bocanegra AR, Rincón Llorente B, Sánchez Hernández E, Benítez Echegoyen F, Borja R, Raposo Bejines F, Colmenarejo Morcillo MF (2008). Batch culture growth of *Chlorella zofingiensis* on effluent derived from two-stage anaerobic digestion of two-phase olive mill solid waste. Electron J Biotechnol.

[CR108] Trummer E, Fauland K, Seidinger S, Schriebl K, Lattenmayer C, Kunert R, Vorauer-Uhl K, Weik R, Borth N, Katinger H, Müller D (2006). Process parameter shifting: Part I. Effect of DOT, pH, and temperature on the performance of Epo-Fc expressing CHO cells cultivated in controlled batch bioreactors. Biotechnol Bioeng.

[CR109] Unrean P, Srienc F (2010). Continuous production of ethanol from hexoses and pentoses using immobilized mixed cultures of *Escherichia coli* strains. J Biotechnol.

[CR110] van Heerden CD, Nicol W (2013). Continuous and batch cultures of *Escherichia coli* KJ134 for succinic acid fermentation: metabolic flux distributions and production characteristics. Microb Cell Factories.

[CR111] Vogel JH, Nguyen H, Giovannini R, Ignowski J, Garger S, Salgotra A, Tom J (2012). A new large-scale manufacturing platform for complex biopharmaceuticals. Biotechnol Bioeng.

[CR112] Wagner B (1987) Leistungsvergleich von Bioreaktoren für mycelförmiges Wachstum. Dissertation, ETH Zürich

[CR113] Wegel S, Heine H, Adelmann S, Schulze-Halberg H (1996). Maßnahmen für den sicheren Umgang mit biologischen Agenzien im Produktionsbereich. Arbeitsschutz in Biotechnologie und Gentechnik.

[CR114] Weir G, Chisholm G, Leveneur J (2020) The magnetic field about a three-dimensional block neodymium magnet. ANZIAM J:1–20. 10.1017/S1446181120000097

[CR115] Werner S, Kaiser SC, Kraume M, Eibl D (2014). Computational fluid dynamics as a modern tool for engineering characterization of bioreactors. Pharm Bioprocess.

[CR116] Werner S, Kraume M, Eibl D (2019) Mixing systems for single-use. In: Eibl R, Eibl D (eds) Single-use technology in biopharmaceutical manufacture. Wiley, Hoboken, pp 25–35

[CR117] Wilke H-P, Weber C, Fries T (1988). Rührtechnik.

[CR118] ZETA GmbH (2020a) Advanced mixing technology. https://www.zeta.com/en/advanced-mixing-technology_235.htm. Accessed 10 Aug 2020

[CR119] ZETA GmbH (2020b) ZETA magnetic agitators. https://www.zeta.com/en/0uploads/dateienEnglisch450.pdf. Accessed 26 Mar 2020

[CR120] ZETA GmbH (n.d.) Bottom-mounted magnetic agitator XXL

[CR121] Zhang J, Gao Z, Cai Y, Cao H, Cai Z, Bao Y (2017). Power consumption and mass transfer in a gas-liquid-solid stirred tank reactor with various triple-impeller combinations. Chem Eng Sci.

[CR122] Zhong JJ (2010). Recent advances in bioreactor engineering. Korean J Chem Eng.

[CR123] Zhu LK, Song BY, Wang ZL, Wang YK (2013). Optimze the structure of impeller for stirred bioreactor. Adv Mater Res.

[CR124] Zlokarnik M (2001). Stirring : Theory and Practice.

[CR125] Zlokarnik M (2006). Scale-Up in Chemical Engineering.

